# pUL36 Deubiquitinase Activity Augments Both the Initiation and the Progression of Lytic Herpes Simplex Virus Infection in IFN-Primed Cells

**DOI:** 10.1128/jvi.00963-22

**Published:** 2022-10-31

**Authors:** Jonas Mohnke, Irmgard Stark, Mara Fischer, Patrick M. Fischer, Andreas Schlosser, Arnhild Grothey, Peter O’Hare, Beate Sodeik, Florian Erhard, Lars Dölken, Thomas Hennig

**Affiliations:** a Institut für Virologie und Immunbiologie, Julius-Maximilians-Universität Würzburg, Würzburg, Germany; b Rudolf-Virchow-Zentrum - Center for Integrative and Translational Bioimaging, Julius-Maximilians-Universität Würzburg, Würzburg, Germany; c Department of Virology, Imperial College London, Norfolk Place, London, United Kingdom; d Institut für Virologie, Medizinische Hochschule Hannover, Hannover, Germany; e RESIST Exzellenzcluster, Medizinische Hochschule Hannover, Hannover, Germany; University of Arizona

**Keywords:** herpes simplex virus 1, interferon, UL36, ubiquitin, DUB, HSV-1, interferon antagonism, USP, innate immunity

## Abstract

The evolutionarily conserved, structural HSV-1 tegument protein pUL36 is essential for both virus entry and assembly. While its N-terminal deubiquitinase (DUB) activity is dispensable for infection in cell culture, it is required for efficient virus spread *in vivo*, as it acts as a potent viral immune evasin. Interferon (IFN) induces the expression of hundreds of antiviral factors, including many ubiquitin modulators, which HSV-1 needs to neutralize to efficiently initiate a productive infection. Herein, we discover two functions of the conserved pUL36 DUB during lytic replication in cell culture in an understudied but equally important scenario of HSV-1 infection in IFN-treated cells. Our data indicate that the pUL36 DUB contributes to overcoming the IFN-mediated suppression of productive infection in both the early and late phases of HSV-1 infection. We show that incoming tegument-derived pUL36 DUB activity contributes to the IFN resistance of HSV-1 in IFN-primed cells to efficiently initiate lytic virus replication. Subsequently, the *de novo* expressed DUB augmented the efficiency of virus replication and increased the output of infectious virus. Notably, the DUB defect was only apparent when IFN was applied prior to infection. Our data indicate that IFN-induced defense mechanisms exist and that they work to both neutralize infectivity early on and slow the progression of HSV-1 replication in the late stages of infection. Also, our data indicate that pUL36 DUB activity contributes to the disarming of these host responses.

**IMPORTANCE** HSV-1 is a ubiquitous human pathogen that is responsible for common cold sores and may also cause life-threatening disease. pUL36 is an essential, conserved herpesvirus protein with N-terminal deubiquitinating (DUB) activity. The DUB is dispensable for HSV-1 replication in cell culture but represents an important viral immune evasin *in vivo*. IFN plays a pivotal role in HSV-1 infection and suppresses viral replication both *in vitro* and *in vivo*. Here, we show that DUB activity contributes to overcoming IFN-induced cellular resistance in order to more efficiently initiate lytic replication and produce infectious virions. As such, DUB activity in the incoming virions increases their infectivity, while the *de novo* synthesized DUB augments productive infection. Thus, the HSV-1 DUB antagonizes the activity of IFN-inducible effector proteins to facilitate productive infection at multiple levels. Our findings underscore the importance of using more challenging cell culture systems to fully understand virus protein functions.

## INTRODUCTION

To limit and suppress viral infection, host organisms have evolved a rich arsenal of antiviral factors, which collectively form the cellular innate antiviral immune system. When cells sense infection via the recognition of viral pathogen-associated molecular patterns (PAMPs) through pattern recognition receptors (PRRs), they activate signaling cascades, which culminate in the interferon regulatory factor (IRF) 3, IRF7, and nuclear factor kappa-light-chain-enhancer of the activated B cell (NF-κB-)-mediated induction and the secretion of type I interferons (IFNs) (1–3). Via autocrine and paracrine activation of the type I IFN receptor (IFNAR), the expression of several hundred IFN-stimulated genes (ISGs), which exert antiviral activity, is stimulated (4–6). The production of ISGs installs an antiviral state in both infected and neighboring, uninfected cells, which hampers susceptibility and permissiveness to productive infection.

Herpes simplex virus 1 (HSV-1) is a double-stranded DNA virus that productively infects a large variety of cell types. IFN-induced effector mechanisms inhibit HSV-1 at various stages of its infection cycle (7, 8) including virus entry (9), the initiation of immediate early gene expression (10–17), and efficient viral protein production (18). To counteract these antiviral effectors, HSV-1 has evolved a plethora of viral immune evasins that efficiently interfere with the innate immune system during lytic infection (reviewed in [19, 20]). Many of the host’s innate defense mechanisms depend on cycles of the ubiquitination/deubiquitination of target proteins to modulate their functions (reviewed in [21, 22]). In turn, HSV-1 usurps the ubiquitin system to counter these defenses through the functions of the viral proteins ICP0 (reviewed in [23]) and pUL36 (see below).

Herein, we characterize the function of the essential large tegument protein pUL36 (also named VP1-2) in the context of an established innate immune response. pUL36 is well-conserved among the different herpesviruses and contributes multiple indispensable functions both early (tegument-associated) and late (free and tegument-associated) in herpesvirus infections (24–29). Upon the entry of a virus particle into a cell, pUL36 remains associated with the incoming capsids to facilitate microtubule-mediated transport toward the nucleus and docking at the nuclear pores (24, 26, 29–37). Late in infection, the pUL36 protein is essential for secondary envelopment and tegument assembly (24, 27, 29, 34). Besides its essential, structural functions, pUL36 and its homologs also comprise an N-terminal deubiquitinating domain (DUB) (38, 39), which, despite its evolutionary conservation, is not essential for HSV-1 infection *in vitro* (40, 41). Interestingly, an N-terminal pUL36 fragment, which comprises the catalytically active DUB domain, can be detected in infected cells at late times of infection (39). On the molecular level, the HSV-1 pUL36 DUB and its homologs are cysteine proteases that, at least in the case of HSV-1, can cleave both K48- and K63-linked polyubiquitin chains (39, 40). As such, DUB activity maintains pUL36 stability by deubiquitinating itself (40, 42). The HSV-1 pUL36 DUB and its homologs in other herpesviruses fulfil a variety of different functions that range from antagonizing the innate immune and DNA damage responses, facilitating appropriate intracellular capsid motility and tegument assembly, and augmenting infection both *in vitro* and *in vivo* (41, 43–53).

Since ubiquitination/deubiquitination cycles are integral to innate immunity signaling (reviewed in [54]), the HSV-1 pUL36 DUB might also interfere with innate immunity at multiple levels: (i) IFN induction, (ii) IFN signaling (after IFNAR activation), and (iii) ISG functions. Indeed, the pUL36 DUB actively contributes to the reduction of the induction of type I IFNs by reversing the ubiquitination of TNF receptor-associated factor 3 (TRAF3) (52), NF-κB inhibitor alpha (IκBα) (55), and the stimulator of interferon genes (STING) (41). Also, pUL36 inhibits type I IFN signaling by binding to the IFNAR independently of its DUB activity (56).

However, its functions during an established IFN response have not yet been characterized. Herein, we show that the HSV-1 pUL36 DUB contributes to disarming IFN-induced antiviral effector mechanisms that interfere with the induction of lytic infection. These effects were already mediated by the pUL36 that entered the host cell cytosol with the incoming capsids. Moreover, we demonstrate that the pUL36 DUB activity was also required later in the infection for the efficient production of infectious progeny virus in IFN-treated cells. Collectively, our data highlight an important role of this HSV-1 DUB in antagonizing the IFN-induced and ubiquitination-mediated cellular defenses, both early and late in an infection.

## RESULTS

### Replication characteristics of parental and pUL36 DUB mutant HSV-1 strains.

**FIG 1:**
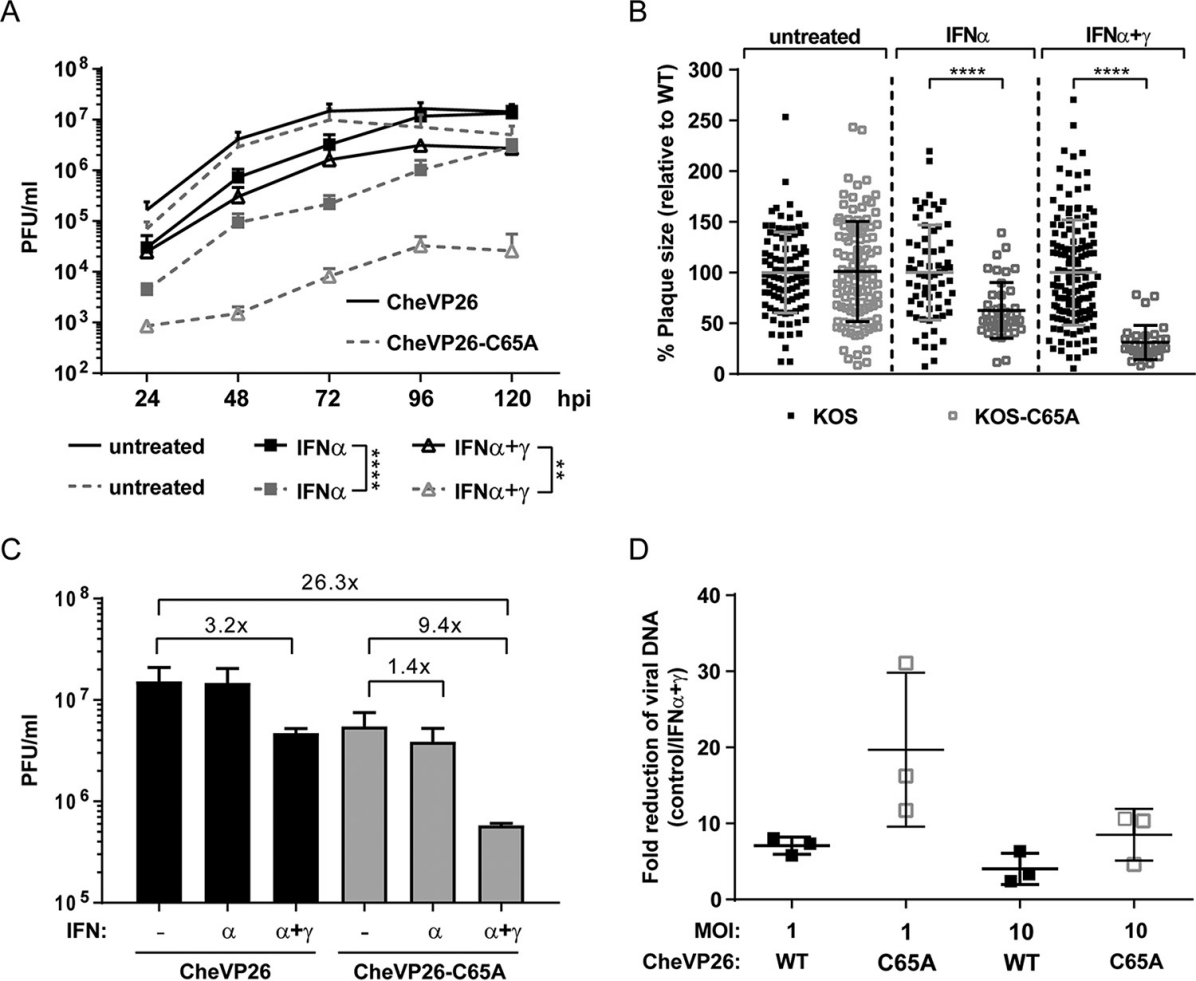
Growth kinetics of parental HSV-1 and its pUL36 DUB mutants. (A) Multistep growth curves of the CheVP26 (black lines) and CheVP26-C65A (gray lines) viruses in untreated (no symbols), 500 IU/mL IFN-α pretreated (filled squares), or 500 IU/mL IFN-α and 100 IU/mL IFN-γ pretreated Vero cells (open triangles) (*n* = 3). IFN was added 20 h prior to inoculation and was replenished after inoculation. Cells were infected with a MOI of 0.001 (untreated and IFN-α) or 0.1 (IFN-α/γ) of either the parental CheVP26 or the CheVP26-C65A virus. The total virus yield was determined in 24 h intervals up to 120 hpi via plaque assay on IFN-naive Vero cells. (B) Plaque size measurements from KOS or KOS-C65A infected Vero cells. The results from a representative experiment are shown. Untreated or IFN pretreated (as in panel A) Vero cells were infected with ~100 PFU/well (100-fold more for IFN-α/γ) and fixed at 2 dpi for untreated cells and at 4 dpi for IFN-treated cells. Plaque sizes were measured using Fiji software. (A and B) Statistical analysis was performed using the Two-Way ANOVA analysis plug-in of GraphPad Prism. (C) Untreated or IFN pretreated Vero cells (500 IU/mL IFN-α alone or in combination with 100 IU/mL IFN-γ) were infected at a high MOI (untreated, MOI of 10; IFN-α, MOI of 20; IFN-α/γ, MOI of 100). The total virus (cells and medium) was harvested at 24 hpi, and the yield was determined via plaque assay on untreated Vero cells. The mean yield from two independent experiments is shown. (D) Untreated or IFN pretreated Vero cells (500 IU/mL IFN-α and 100 IU/mL IFN-γ) were infected for 1 h, and replicated viral DNA was measured at 6 hpi. The mean fold reduction of replication was calculated via the ΔΔCq method, using β-actin as a cellular reference. **, *P* < 0.01; ****, *P* < 0.0001.

Despite its important role in the spread of HSV-1 in animals, the DUB of pUL36 contributes little to productive infection in cell culture (40, 41). Thus, we hypothesized that it might prevent the shutdown of viral infection in the presence of preestablished or ongoing innate immune responses as they induce the expression of the many host proteins that are involved in ubiquitin-mediated pathways (Interferome database v2.0 [57]). Thus, we tested whether pretreatment with type I IFNs or with both type I and II IFNs affected the multicycle growth kinetics of the catalytically inactive DUB mutant HSV1-(17^+^)Lox-CheVP26-UL36C65A (CheVP26-C65A) in comparison to its parental HSV1-(17^+^)Lox-CheVP26 (CheVP26) virus. As previously reported (40, 41), there was little difference in viral yield from the different cell lines for both viruses in the absence of IFN (data not shown). Consistent with previous reports (8, 58), there was a modest reduction in the yield of CheVP26 from IFN-α pretreated Vero cells (Fig. 1A). A greater inhibition was attained by combining type I and II IFNs (Fig. 1A) as reported earlier (7). Notably, since plaque formation on IFN-α/γ pretreated Vero cells of wild-type HSV-1 dropped up to 100-fold, we performed inoculations with 100-fold higher virus doses for the IFN-α/γ combination treatment (i.e., a multiplicity of infection [MOI] of 0.1 for the IFN-α/γ pretreated Vero cells and a MOI of 0.001 for untreated or IFN-α pretreated Vero cells). An enhanced sensitivity of the C65A mutant to IFN pretreatment was already apparent prior to harvest, as the cytopathic effect (CPE) with CheVP26-C65A was much reduced compared to that of the parental CheVP26 virus (data not shown). This translated into a more pronounced reduction of the virus yield of the mutant compared to the parental virus as early as 1 day postinfection (dpi) with either treatment (Fig. 1A). At 3 to 5 dpi, IFN-α pretreatment reduced the yield of the C65A mutant compared to that of the parental CheVP26 virus by approximately 10-fold, and this increased to roughly 100-fold upon combined IFN-α/γ treatment (Fig. 1A). In agreement with this result, infection with another DUB mutant (KOS-C65A) of Vero that had been pretreated with IFN-α alone or in combination with IFN-γ led to a 30% or 70% reduction in plaque sizes compared to those of the parental KOS virus, respectively (Fig. 1B). We also noted a modest but reproducible reduction in plaque numbers for both DUB mutants, compared to their respective parental viruses, on IFN pretreated cells.

Next, we investigated whether the drop in yield after multicycle replication and the reduction in the plaque sizes of the DUB mutants were due to the reduced or delayed production of infectious virions. Thus, we performed high MOI infections of Vero cells and analyzed virus yields at 1 dpi (at a MOI of 10 for untreated cells, a MOI of 20 for cells pretreated with IFN-α, and a MOI of 100 for cells pretreated with both IFN-α and IFN-γ) (Fig. 1C). As explained above, initial experiments had indicated that pretreatment with IFN-α alone reduced infectivity mildly but that the combination of IFN-α and IFN-γ reduced plaque formation between 30 and 100-fold (data not shown) and necessitated an increased inoculation dose to ensure that, irrespective of the IFN treatment regimen, all cells had been synchronously infected. In the absence of IFN pretreatment, there was a mild reduction in yield for the CheVP26-C65A mutant (2.8-fold) compared to its parental virus (Fig. 1C). While IFN-α had little effect on the parental CheVP26 virus, it led to a 1.4-fold drop in the yield of the C65A mutant. The combined pretreatment with IFN-α and IFN-γ reduced the yield of the parental virus by 3.2-fold and that of the C65A mutant by 9.4-fold. This reduction of infectious virus coincided with a more pronounced inhibition of viral DNA replication for the DUB mutant, measured at 6 h postinfection (hpi) in Vero cells treated with a combination of IFN-α and IFN-γ (Fig. 1D). Collectively, these data indicate that pUL36 DUB activity is required for efficient DNA replication and progeny production in IFN pretreated cells, which explains the strong replication defect of the DUB mutants in multistep growth experiments.

### DUB activity is required for plaque initiation in IFN pretreated cells.

**FIG 2:**
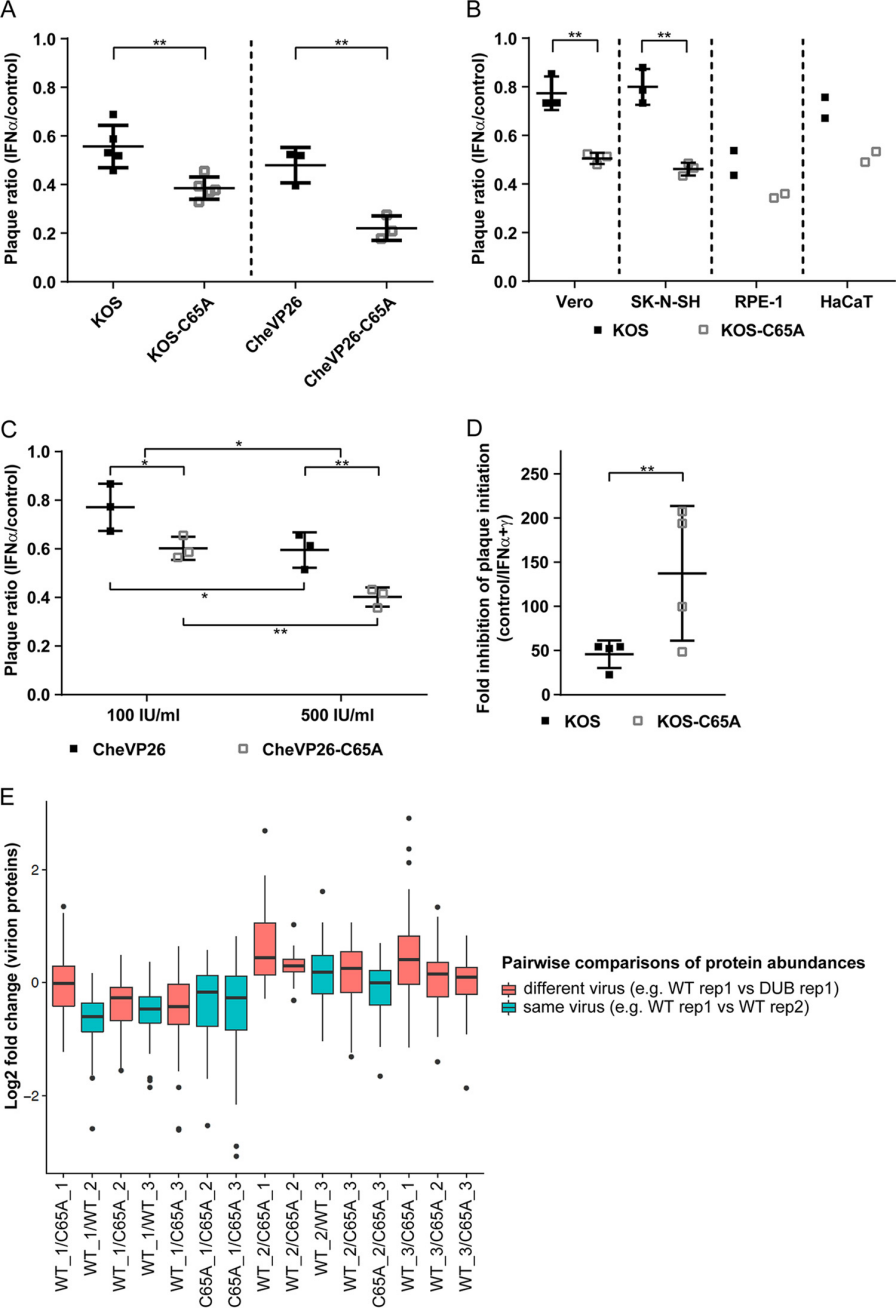
Effect of the pUL36 DUB on plaque initiation. (A–D) Vero cells were either left untreated or pretreated with different regimens of IFN-α either alone (A–C) or in combination with IFN-γ (D). Plaque assays were performed 20 h after the treatment of the cells with IFN using ~100 PFU/well (A–D) or 10,000 PFU/well (panel D, only for the IFN-α/γ treated wells) of either parental or isogenic C65A mutants (strains indicated). The ratio of plaques that formed in the presence and absence of IFN (panels A–C, IFN-α/control) or the fold inhibition of plaque formation (D, control/IFN-α/γ) is shown. (B) Using the experimental setup employed in panel A with minor modifications (see Materials and Methods for the IFN-α concentrations used), the plaque formation was compared on untreated and IFN-α pretreated Vero and human SK-N-SH, HaCaT, and RPE-1 cells. (A–D) Statistical analysis was performed using the One-Way ANOVA analysis plug-in of GraphPad Prism. (E) Mass spectrometric analysis of three independently generated gradient purified virion preparations of the CheVP26 and CheVP26-C65A viruses (parental and mutant virus prepared as pairs on the same day). Differences in the identified virion components were compared between the different biological replicates (i.e., virion preparations) of the same (turquoise) and different (red) viruses. All abundances were normalized to VP5 content, and only viral proteins are shown. *, *P* < 0.05; **, *P* < 0.01; ns, not significant.

Since IFN pretreatment had reduced the numbers of plaques formed by the DUB mutant, we next investigated whether pUL36 DUB activity plays any role in the disarming of IFN-induced host defense mechanisms that act immediately upon virus entry and thereby affect specific infectivity or cell susceptibility. To test this, we compared the efficiency of plaque initiation (i.e., the number of plaques forming with a given inoculum) for the parental and their respective DUB mutant viruses in IFN-α-treated and untreated cells. Notably, both the IFN-α-treated wells and the untreated wells were infected with the same virus inoculum within minutes of each other. We divided the number of plaques in the IFN pretreated cells by that of the untreated cells to express the effect of IFN pretreatment as a relative plaque ratio [IFN/control]. A ratio of 1 would indicate that a given inoculum gave rise to the same number of plaques on untreated and IFN pretreated cells. A ratio of 0 would indicate that the IFN pretreatment completely abolished plaque formation. In comparison to growth curves, which measure differences that are affected by many factors at potentially every step in the virus life cycle and mostly quantify phenotypes in log scale, the plaque ratio assay only measures the formation of plaques, not the sizes of plaques or other replication parameters. Thus, even subtle phenotypic differences can be accurately quantified on a linear scale. We use this measure for the following analyses to express phenotypic differences between the parental and their respective DUB mutant viruses. In the first set of experiments, we compared two HSV-1 UL36-C65A mutants of two HSV-1 BAC-derived strains, KOS and CheVP26 (strain 17). Expectedly, IFN-α induction with 500 IU/mL decreased the number of plaques obtained with both parental viruses by about 1.5 to 2-fold (Fig. 2A). This means that IFN-α pretreatment almost doubled the perceived particle-to-PFU ratio of both viruses. A similar effect was seen on multiple cell lines of human origin (Fig. 2B). Moreover, higher doses of IFN led to a more pronounced repression of plaque initiation, with the combination of IFN-α and IFN-γ showing the greatest effect (Fig. 2C and D). Importantly, IFN pretreatment impaired the plaque formation of both DUB mutants significantly more strongly than those of their respective parental strains (*P* < 0.01), consistent with a further increase in the perceived particle-to-PFU ratio of the DUB mutants of between 2.5 to 4-fold (Fig. 2A). Consistently, we observed a similar reduction of the plaque initiation of the KOS-C65A mutant on human keratinocyte (HaCaT), retinal pigment epithelial (RPE-1), and neuroblastoma (SK-N-SH) cell lines (Fig. 2B), which were treated with an IFN-α dose that induced comparable inhibitory effects against wild type HSV-1 on the different cell lines.

Since IFN pretreatment affected infectious virus production of the DUB mutant (Fig. 1A and C), we considered that the lack of DUB activity might affect virion assembly of the virus stocks of the DUB mutant. For this, we characterized the protein composition of three gradient-purified virion preparations of the parental and DUB mutant viruses (CheVP26), including the ones used for this study, via quantitative mass spectrometry (6-plex TMT). We detected 54 viral proteins being packaged in comparable quantities, including VP5, pUL36 (including the mutated peptide of the C65A mutant), VP22, VHS, and pUL25, as well as the pUL36 binding proteins pUL37 and VP16. In fact, the variance of protein abundances between different preparations of the same virus (Fig. 2E, blue boxes; e.g., replicate 1 versus replicate 2 of CheVP26) did not differ much from the variance between the preparations of the parental and DUB mutant viruses (Fig. 2E, red boxes; e.g., replicate comparison of CheVP26 versus CheVP26-C65A). Additionally, there were no obvious differences in the appearance of the Ficoll gradients of the different stock preparations (data not shown), unlike that which was previously described for a ΔICP22 mutant (59). The particle-to-PFU ratios of the parental and DUB mutant virus stocks used in this study were also similar (data not shown). Together these data indicate that the lack of pUL36 DUB activity did not measurably affect the protein composition of the DUB mutant virus stocks.

**FIG 3:**
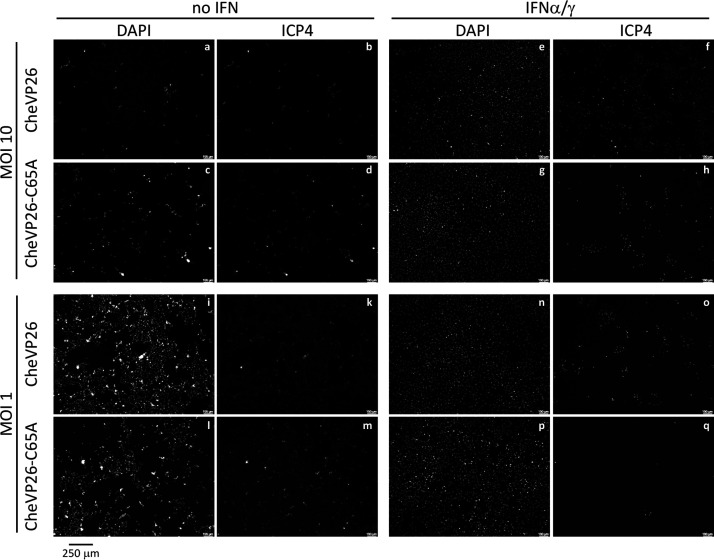
Effect of the DUB mutation on infectivity at high MOI. Untreated (no IFN) or IFN-α/γ pretreated Vero cells were infected with serially diluted inocula of either the CheVP26 or the CheVP26-C65A virus. MOIs of 10 and 1 are shown. The cells were fixed at 24 hpi and stained with anti-ICP4 antibody in order to detect infected cells. The cellular nuclei were stained with DAPI. Grayscale images with enhanced brightness and contrast are shown. Scale bars are indicated on the bottom right of each image. For clarification, a scale bar of 250 μm (identical for each image) was added underneath the figure.

Next, we tested whether the DUB-mediated effects on plaque formation occurred only at low MOIs, as HSV-1 mutants lacking expression of another tegument component, ICP0, also exhibit a much stronger inhibition at low MOIs (60). For this, we infected control or IFN-α/γ pretreated Vero cells with serially diluted inocula of CheVP26 or CheVP26-C65A for 24 h (Fig. 3). We detected infected cells by staining with an antibody against ICP4 to ensure the detection of even small infectious centers. After the infection of untreated cells at a MOI of 10, CPE was almost complete for the parental and the C65A mutant viruses (Fig. 3A–D). In contrast, the monolayer of IFN-α/γ pretreated Vero cells was still almost intact (Fig. 3E–G). However, inspection of the ICP4 staining revealed that the C65A mutant had infected fewer cells than had the parental virus (Fig. 3, panels F and H). Similar trends were obtained upon infection with a MOI of 1. Again, both viruses showed that all of the untreated cells were expressing ICP4 by 24 hpi and that the monolayer was not fully intact (Fig. 3I–M). In contrast, the parental CheVP26 virus showed a far more advanced infection with more and larger infected cell clusters of the IFN-α/γ pretreated cells (Fig. 3N–Q). Collectively, these data show that DUB activity is required for full infectivity at low and high MOI values. This is consistent with a role of the pUL36 DUB early during infection, possibly while still being associated with the capsids or after the DUB domain has been cleaved from the incoming capsids.

### pUL36 DUB promotes the efficient initiation of infection independently of its effect on IFN signaling.

**FIG 4:**
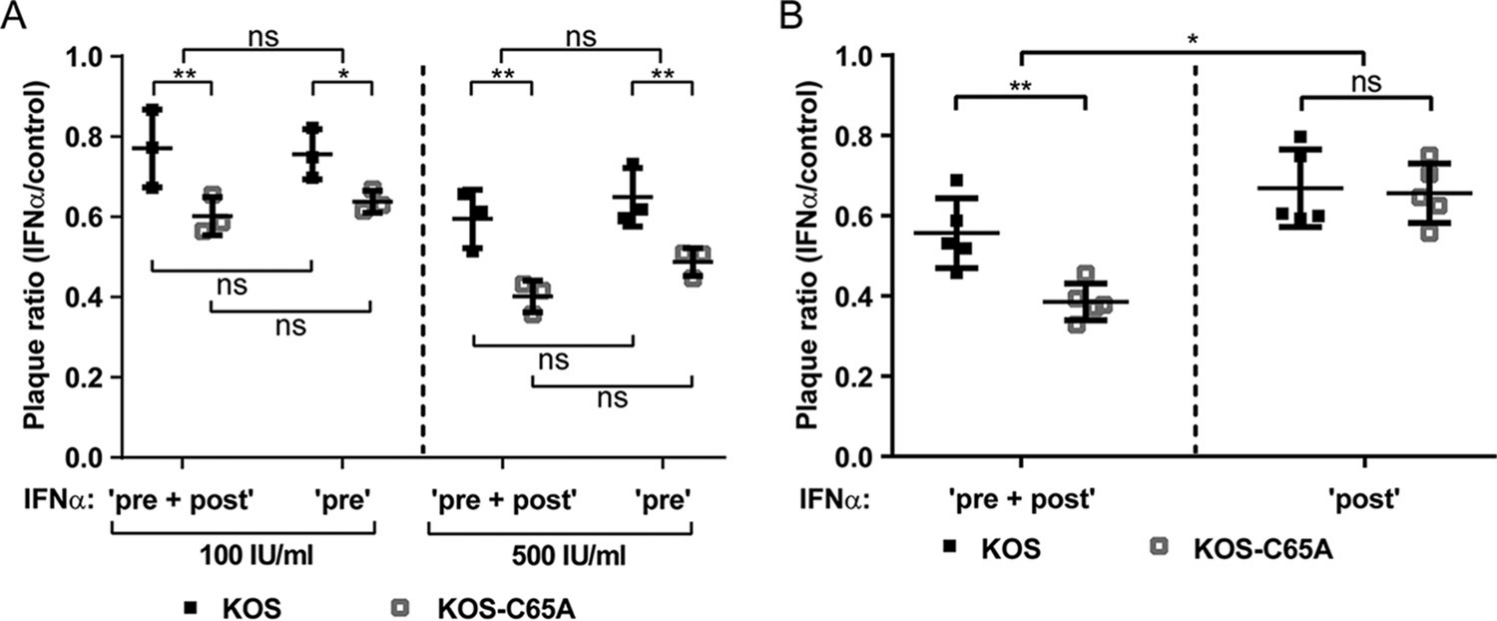
The UL36 DUB augments plaque formation in IFN pretreated cells. (A) Vero cells, which were left untreated, pretreated with 100 or 500 IU/mL IFN-α for 20 h prior to inoculation only (“pre”), or treated with 100 or 500 IU/mL IFN-α prior to and after inoculation (“pre + post”), were infected with ~100 PFU/well of the KOS or KOS-C65A viruses. (B) Similar to panel A, except that 500 IU/mL IFN-α treatment was applied throughout the whole assay (“pre + post”) and was compared to treatment with 500 IU/mL IFN-α applied only after inoculation (“post”). (A and B) Plaque ratios of IFN-treated to IFN-untreated Vero cells were calculated and plotted. Statistical analysis was performed using the Two-Way ANOVA analysis plug-in of GraphPad Prism. *, *P* < 0.05; **, *P* < 0.01.

HSV-1 pUL36 DUB inhibits IFN signaling by binding to the IFNAR, thereby interfering with the induction of ISG expression (56). As we had pretreated cells with IFN prior to inoculation, it seemed unlikely that plaque formation was reduced due to pUL36 inhibiting any IFN signaling. Nevertheless, we determined whether the DUB activity also promoted the infection of cells treated with IFN-α after virus inoculation. For these experiments, Vero cells were infected with the KOS-C65A mutant or the parental KOS virus after they had been (i) treated with 100 or 500 IU/mL IFN-α only prior to inoculation (“pre”), (ii) treated prior to and after inoculation (“pre + post”), or (iii) treated only after inoculation (“post”). Interestingly, IFN pretreatment alone (pre) was as good at inhibiting plaque formation as was treatment throughout (pre + post) for the parental KOS virus. Also, reduced plaque formation of the DUB mutant was similarly efficient with both treatment regimens (Fig. 4A). In contrast to the continuous treatment (pre + post), IFN treatment only after inoculation (post) did not differentially inhibit the plaque formation of the DUB mutant but rather showed a modest inhibition for both the DUB mutant and its parental KOS virus (Fig. 4B). Taken together, these data indicate that HSV-1 requires a small amount of pUL36 DUB protein to be delivered to the cytosol in order to efficiently initiate an infection in cells which have already established an IFN-induced antiviral state.

### Tegument-derived pUL36 DUB activity is required to overcome IFN-induced antiviral restriction.

**FIG 5:**
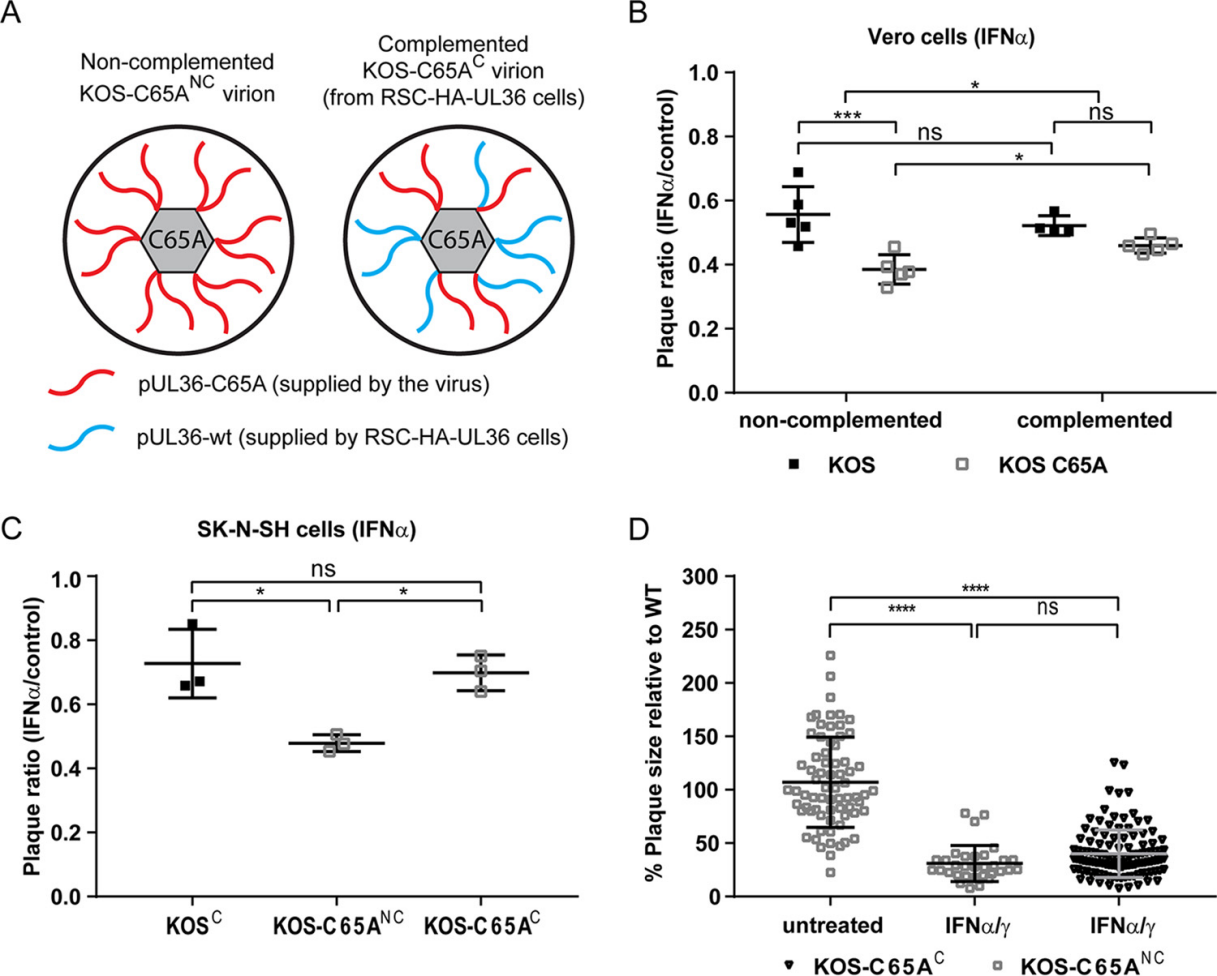
Tegument UL36 DUB activity augments plaque initiation in IFN pretreated cells. (A) Schematic representation of non-complemented DUB mutant virions (left panel) and complemented DUB mutant virions (right panel). When the C65A virus is grown on normal producer cells (those not expressing pUL36, e.g., RS cells) the virions, which are produced, only contain the mutant, virus-derived pUL36 protein (red). When the C65A virus is grown on UL36 complementing cells (e.g., RSC-HA-UL36), the resulting virions contain a mixture of mutant, virus-derived pUL36 and wild-type, cell-derived pUL36 (blue, right panel). Parental virus grown on the respective cells served as controls. After the initial infection of noncomplementing cells (e.g., Vero or SK-N-SH cells) with complemented DUB mutant, the plaques progressed as “mutant”, since the virions produced in these cells only contain *de novo* synthesized pUL36-C65A. (B and C) Vero or SK-N-SH cells were treated prior to and after inoculation with (B) 500 IU/mL IFN-α or (C) 25 IU/mL IFN-α and were infected at ~100 PFU/well with the indicated viruses. Complementation status is indicated as “C” (complemented) or “NC” (non-complemented). Plaque ratios were calculated and plotted as before. (D) Plaque sizes on untreated and IFN-α/γ pretreated Vero cells were analyzed for the complemented DUB mutant virus and compared to that of the non-complemented virus. Each data point represents one plaque which was normalized against the mean plaque size of the parental virus assayed simultaneously. (B–D) Statistical analysis was performed using the (C and D) One-Way ANOVA or (B) Two-Way ANOVA analysis plug-in of GraphPad Prism. *, *P* < 0.05; ***, *P* < 0.001; ****, *P* > 0.0001; ns, not significant.

To test this hypothesis, we isolated HSV-1 virions for both the parental KOS and the KOS-C65A mutant from RSC cells or complementing RSC-HAUL36 cells (Fig. 5A). The RSC-HAUL36 cell line enables pUL36 expression from the authentic HSV-1 UL36 promoter upon infection and efficiently rescues the infectivity of HSV-1 mutants impaired in any of the documented pUL36 functions (27, 29, 30, 34, 61). It is expected that the virions of the parental KOS virus contain regular wild-type pUL36 protein, irrespective of the cell line used for amplification. In contrast, the virions of the KOS-C65A mutant prepared from RSC cells will contain exclusively pUL36 proteins lacking the DUB activity (pUL36-C65A) (red in Fig. 5A), and the virions prepared from the complementing cells will contain a mixture of wild-type pUL36 expressed from the RSC-HAUL36 host genomes and of pUL36-C65A expressed from the viral genomes.

For this series of experiments, IFN-α pretreated or control Vero cells were again inoculated with 100 PFU/well of the indicated viruses (Fig. 5B). Interestingly, virions containing only pUL36-C65A proteins (non-complemented KOS-C65A) (Fig. 5B), were more impaired by the IFN pretreatment and generated lower plaque ratios (>50%; IFN/control) than those complemented with wild-type pUL36 proteins (complemented KOS C65A) (Fig. 5B) in IFN-α pretreated Vero cells. More strikingly, on SK-N-SH cells pretreated with IFN-α, the complemented KOS-C65A^C^ (Fig. 5C) formed as many plaques as did the complemented parental virus (KOS WT^C^) (Fig. 5C). While the plaque ratio of the KOS-C65A mutant was partially rescued via transcomplementation (complemented KOS-C65A in Fig. 5B; KOS-C65A^C^ in Fig. 5C), this complementation did not increase the plaque sizes in IFN pretreated, non-complementing Vero cells compared to those of the KOS-C65A mutant virions prepared in non-complementing cells (KOS-C65A^C^ versus KOS-C65A^NC^ in Fig. 5D). These data indicate that the transcomplementation had only affected the initial infection of the first cell which gave rise to a plaque and that the small amount of incoming pUL36 DUB derived from the transcomplemented inoculum enabled the HSV-1 DUB mutant to overcome some IFN-induced, antiviral effector mechanisms, which work to prevent plaque initiation.

### IFN withdrawal does not rescue the infectivity of the DUB mutant.

**FIG 6:**
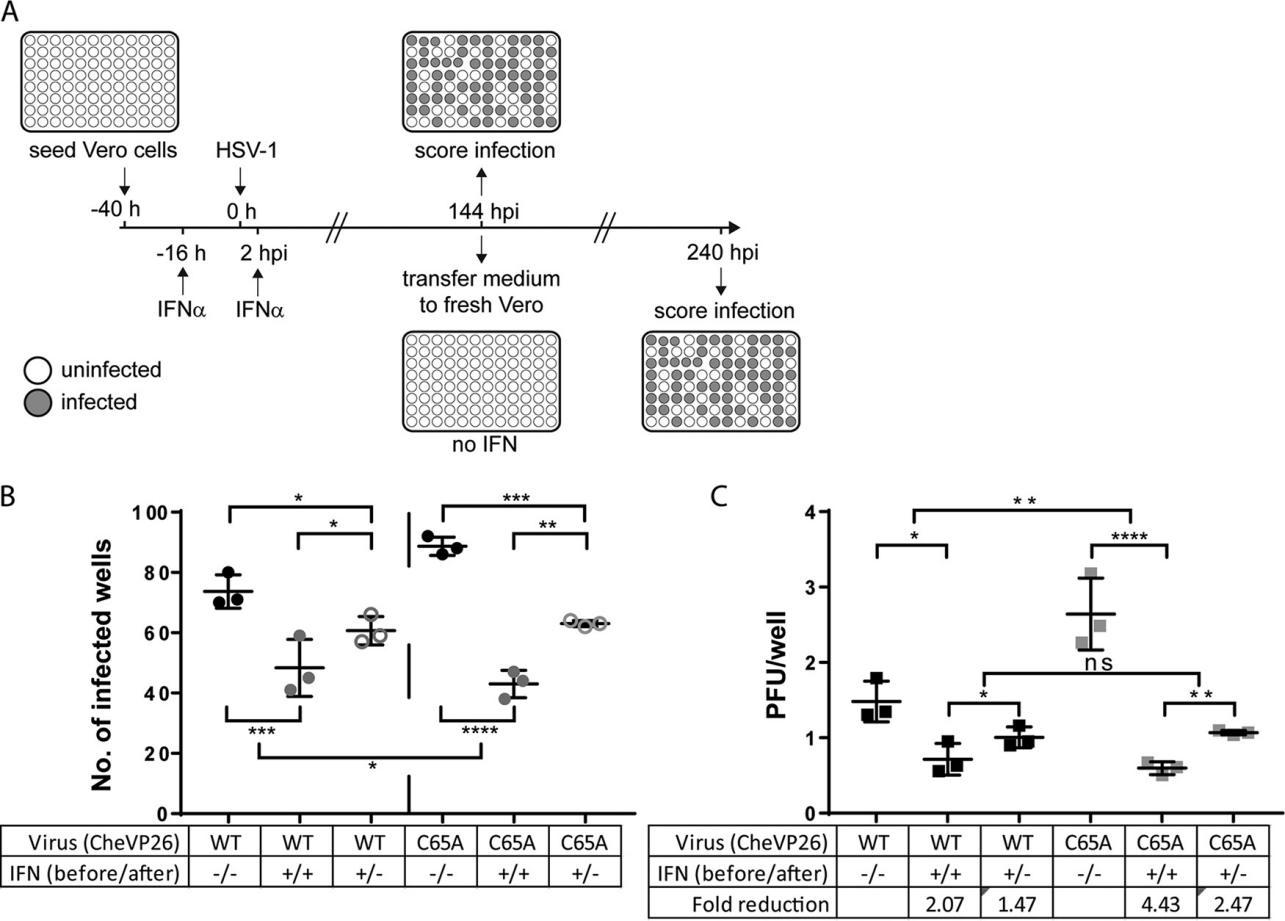
IFN-mediated inhibition of plaque initiation is partially irreversible and greater for the C65A mutant. (A) Schematic of the experimental conditions. Vero cells were either left untreated (−/−) or treated with 500 IU/mL IFN-α, both prior to and after infection (+/+). To check for the reversibility of the IFN-induced inhibition of plaque initiation, we also included a third condition, in which IFN was removed from the infected cells immediately prior to virus inoculation (+/−). The cells were infected with approximately 2 PFU/well of the CheVP26 or CheVP26-C65A viruses. At 6 dpi, infected wells were scored via fluorescence microscopy (mCherry, indicated as gray wells). Additionally, medium from each well was transferred to untreated Vero cells to ensure the detection of even low levels of productive virus replication. After an additional 4 days, the infected wells (mCherry-positive) were scored via fluorescence microscopy and via crystal violet staining. (B) The numbers of infected wells per 96-well plate from the three independent experiments are shown. Only scores obtained after the full 10 days are shown. (C) Extrapolated multiplicity of infection of the data presented in panel B. Based on the percentage of infected wells, the infectivity of the virus inoculum (in PFU/well) under the respective treatment conditions was determined using a Poisson distribution and plotted for each virus and treatment condition. The fold reduction was calculated based on the infectivity of the respective virus on untreated Vero cells (control/IFN-α). (B and C) Statistical analysis was performed using the Two-Way ANOVA analysis plug-in of GraphPad Prism. *, *P* < 0.05; **, *P* > 0.01; ***, *P* < 0.001; ****, *P* > 0.0001; ns, not significant.

We previously reported that IFN pretreatment efficiently suppresses murine cytomegalovirus (MCMV) infection by preventing the expression of viral immediate early genes in a PML body-dependent manner (62). Upon IFN withdrawal, MCMV gene expression resumes, and efficient lytic infection is reinitiated. Since we discovered here that IFN pretreatment reduced plaque initiation of the UL36-C65A mutant, and since IFN protection against HSV-1 is known to wane within 24 h of treatment (63), IFN withdrawal at the time of inoculation could ultimately lead to the reactivation of HSV-1 genomes that had been reversibly repressed by induced IFN effector proteins. To test this hypothesis, we pretreated Vero cells cultured in 96-well plates for 16 h with 500 IU/mL of IFN-α and then infected them with either CheVP26 or the CheVP26-C65A mutant at 1.5 PFU/well for 6 days in the absence (pre) or the presence (pre + post) of IFN-α (Fig. 6A). We had determined beforehand that an infection with a low infectious dose of 1.5 PFU/well gave rise to about 77% of infected wells on average (data not shown). At 6 dpi, the supernatants were transferred to untreated, fresh Vero cells to detect any infectious virus that had been released from the first round of inoculated cells. Based on the number of infected wells, we calculated an inoculation dose of 1.5 PFU/well for the CheVP26 virus and an inoculation dose of 2.6 PFU/well for the CheVP26-C65A mutant (Fig. 6B). Importantly, and as suggested by the plaque reduction assay (compare Fig. 6B and C to Fig. 2A), continuous IFN-α treatment (including pretreatment) reduced the infectivity of the CheVP26 virus by about 2-fold from 1.5 to 0.7 PFU/well, whereas that of the DUB mutant was reduced by 4.4 fold from 2.6 to 0.6 PFU/well (compare ‘−/−‘ to ‘+/+’ in Fig. 6C). When IFN was removed after inoculation, the infectivity of the parental and of the C65A mutant increased somewhat (Fig. 6C, ‘+/−‘), although, notably, it did not fully recover. The recovery of the CheVP26-C65A mutant was only marginally greater than that of its parental CheVP26 virus, despite the significantly stronger IFN-α-mediated suppression. These findings are consistent with the hypotheses that IFN induces distinct antiviral effector mechanisms which both irreversibly and reversibly suppress the early processes of HSV-1 infection and that the small amount of incoming pUL36 DUB activity contributes to overcoming a currently unknown irreversible, IFN-induced, antiviral mechanism.

## DISCUSSION

Ubiquitination and deubiquitination are dynamic posttranslational protein modifications that regulate almost every physiological cellular process, including innate immunity (reviewed in [21, 22]). Not surprisingly, HSV-1 has evolved means by which to modulate and counteract these regulatory circuits to promote infection. While the functions of the HSV-1 E3 ligase ICP0 have been studied extensively (recently reviewed in [64]), the role of the HSV-1 pUL36 DUB domain is less understood, due to the fact that HSV-1 pUL36 DUB mutants revealed no impairment in cell culture (40, 41). However, the high degree of conservation of the DUB sequence (38, 39), its position within the herpesvirus genomes and virions, and recent evidence on DUB-dependent immune counterregulation (41, 52, 55, 56) suggest important functions of the HSV-1 DUB in the virus life cycle and pathogenesis. The latter is underscored by phenotypes of other herpesvirus DUB mutants *in vitro* and *in vivo* (42, 46–48, 51, 53).

Herein, we show that in IFN pretreated cells, the HSV-1 pUL36 DUB contributes an important function to disarm antiviral mechanisms. We describe two separate functions wherein the activity of incoming pUL36 DUB contributes to overcoming IFN-induced resistance mechanisms to establish a productive infection. Later on, *de novo* synthesized pUL36 DUB augments the production of viral DNA and infectious virions. The combined activity of incoming and newly synthesized DUB explains the significant reduction of DUB mutant virus replication in the multicycle infection of IFN-primed cells.

IFNs are key regulators of lytic HSV infection *in vivo*, and infected cells sense HSV through a variety of cellular sensors at multiple steps of the viral infection cycle, which is highlighted in several studies (reviewed in [65–68]). *In vivo*, HSV-1 induces IFN production in infected keratinocytes (69) and in the brainstem (41). As such, patients and mice lacking the ability to properly produce or respond to IFN are more sensitive to HSV-1 infections (reviewed in [65]). Importantly, a HSV-1 DUB mutant showed increased viral titers in the brainstems of mice that lack STING function (41). In cell culture, IFN treatment induces strong HSV-1 resistance in nonneuronal cells (e.g., skin cells) and in bone marrow derived dendritic cells (7, 58, 70–72). Neurons are less sensitive to IFN, but higher dose treatments still inhibit HSV-1 replication, albeit at comparably lower levels (70, 71). In a typical *in vivo* setting, the initial infection of the host in the first cell(s) may proceed unimpeded (i.e., without IFN preexposure). The surrounding keratinocytes, dermal fibroblasts, and neurons will subsequently become activated by IFN (as suggested in [73]). Thus, the reduced IFN resistance of DUB mutants that we observed *in vitro* may explain at least part of the described *in vivo* phenotypes and also highlights the importance of DUB function in pathogenesis.

We have shown in complementary assay systems using IFN-primed cells that the DUB augments viral DNA replication and the production of infectious virions. This novel function in IFN pretreated cells is perhaps unsurprising, as IFN induces hundreds of effector proteins (74, 75), including many modulators of the ubiquitin system (see the Interferome database [57]). Several HSV-1 and host proteins undergo cycles of ubiquitination and deubiquitination during infection (17, 42, 64, 76). The DUB might regulate such dynamic ubiquitination states to modulate the functioning of proteins. As such, the DUB affects the ubiquitination status of several cellular innate sensors (41, 52, 55). In addition, the DUB modulates the ubiquitination status of several viral components (40, 42), which could in turn have implications at later stages by ensuring the appropriate expression and function of viral proteins in replication and assembly.

Additionally, we describe a second function of the DUB to promote the initiation of infection at both low and high MOIs in IFN-primed cells. The phenotype of the DUB mutant could be explained by (i) a defect in transport, (ii) a decreased stability of incoming capsids, or (iii) a silencing of the deposited viral genomes in the nucleus. While our data cannot distinguish between these three mutually nonexclusive possibilities, in light of previous work, they provide valuable starting points for further investigations.

Previous reports highlight that herpesvirus capsid stability during entry appears to be a critical factor for timely uncoating at the nuclear pore, as capsids bind to several IFN-inducible E3 ligases (77) and may be targeted for proteasomal degradation both in the cytosol (50, 78) and by the IFN-inducible GTPase MxB (9, 77, 79). In fact, Horan et al. show that the major capsid protein VP5 is ubiquitinated in macrophages (78), although the list of ubiquitinated HSV-1 proteins also includes pUL36, pUL37, pUL25, and pUL6, which were found to be more ubiquitinated in infections with a HSV-1 pUL36 DUB mutant (42). The inner tegument and capsid components pUL36, pUL37, VP5, pUL6, and pUL25 are prime targets for modulation by ubiquitination in the early stages of infection, as these are all still present when capsids dock at the nucleus (29, 34, 80) (also reviewed in [37]). pUL36 itself is likely a highly relevant ubiquitination substrate, as it possesses four ubiquitination sites and auto-catalytic activity (40, 42). Moreover, it is ubiquitinated during assembly or upon subsequent entry into new cells (42). Such ubiquitination events could alter the transport or stability of incoming capsids. The augmentation of plaque initiation observed with the transcomplemented DUB mutant stocks (Fig. 5B and C) could indicate that the DUB functions during entry as a part of the capsid assembly, where local DUB concentrations are high and its effects would be maximal.

In pseudorabies virus, a pUL36 DUB mutant exhibits an assembly defect (48). Thus, to test whether the HSV-1 pUL36 DUB causes alterations to virion composition, we compared several parameters of the DUB mutant to those of parental virus stocks. We found that the particle-to-PFU ratios and protein contents of the parental and DUB mutant virions remained unchanged. Additionally, since the DUB was shown to inhibit IFN production, we used BHK cells for the production of virus stocks, which do not produce IFN (81, 82). Taken together, our data are thus consistent with the hypothesis that the DUB augments the infectivity of HSV-1 in IFN treated cells directly and not through changes to virion composition.

Several studies indicate that HSV-1 genomes deposited into the nucleus of IFN pretreated cells cannot efficiently initiate transcription (83, 84). This restriction is exacerbated in HSV-1 mutants that lack the viral E3 RING ubiquitin ligase ICP0 (58, 72, 85, 86). The N-terminal pUL36 DUB domain is cleaved off of incoming capsids in the cytosol or at the nuclear pores (87) and thus could act to potentially alleviate the ubiquitin-mediated inhibition of infection in the nucleus. Thus, incoming HSV-1 genomes might be silenced more efficiently in the absence of this cleaved DUB activity, possibly through the activity of nuclear, cellular E3 ligases, such as RNF8, as was suggested for the HSV-1 mutants lacking the E3 ubiquitin ligase ICP0 (88, 89).

Our group previously observed that treatment with IFN-β reversibly silenced MCMV infection in endothelial and fibroblast cells (62). In contrast, Suzich et al. (2021) reported nonreversible suppression in IFN-treated neurons, in which PML NBs appeared to entrap the HSV-1 genomes (90). Interestingly, we found that there are both reversible and nonreversible effects of IFN on HSV-1 infectivity, although the DUB did not contribute a reversible effect of the IFN-induced perturbation of HSV-1 infection. Further experimentation is required to investigate the role of PML NBs in the DUB mutant phenotype.

In summary, we have shown for the first time that the HSV-1 pUL36 DUB contributes at least two functions in cells that have been exposed to IFN prior to infection. Our data collectively highlight a physiologically important and thus far underappreciated role of the DUB in modulating virus-host interactions and thereby prime further studies of DUB functions in *in vivo* systems.

## MATERIALS AND METHODS

### Cells.

Vero (Green Monkey kidney epithelial), rabbit skin cells (RSC), RSC-HAUL36 (27), retinal pigment epithelial (RPE)-1, SK-N-SH (human neuroblastoma), and HaCaT (human keratinocytes) were cultured at 37°C and 5% CO2 in a humidified atmosphere in DMEM supplemented with 10% FBS, 1× Penicillin-Streptomycin, and 1× nonessential amino acids. 500 μg/mL Geneticin was added to the medium to grow the RSC-HAUL36.

### Viruses.

We used the HSV-1 KOS-37 BAC (bacterial artificial chromosome, henceforth called KOS) and its derivative KOS UL36-C65A, which harbored an inactive DUB (henceforth KOS-C65A) (40, 91) as well as the HSV1(17^+^)Lox-CheVP26 BAC (henceforth CheVP26), in which the small capsid protein VP26 was tagged with mCherry, and its derivative HSV1(17^+^)Lox-CheVP26-pUL36ΔDUB (29, 41) (henceforth CheVP26-C65A), which contained the UL36.C65A mutation. The C65A mutation in UL36 was inserted into the UL36 gene via *en passant* mutagenesis (92) using the primers listed in Table 1.

**TABLE 1:** Primers used to generate HSV-1(17^+^)Lox-CheVP26-UL36.C65A

Primer ID	Sequence (5′ → 3′)	Comments
prW534	GTATATCTGGCCCGTACATCGATCT	First PCR (*En passant* cloning)
prW853	CATCAAATATGAGGCTGAGAAAGGACAGCGACGAGCGCATCGCCGATACCGACCCCCCCGGCTTAGGGATAACAGGGTAATCGATTT	
prW854	CATCAAATATGAGGCTGAGAAAGGAC	Second PCR (*En passant* cloning)
prW855	CCAGTTCGCGCCCGACCTGGAGCCGGGGGGGTCGGTATCGGCGATGCGCTCGTCGCTGTCCTTGTATATCTGGCCCGTACATCGATCT	
prW902	CGACCAAACATCCCTCGAT	PCR for sequencing
prW429	GGCACATATGATCGCGGG	

A kanamycin resistance cassette flanked by two I-SceI restriction sites was lifted from the pEP-Kan-S vector (93) using primers prW534 and prW853 as well as prW854 and prW855 with two consecutive polymerase chain reactions (PCRs). 100 ng of the gel-purified PCR product from the second PCR were transformed into GS1783 cells containing the HSV1(17^+^)Lox-CheVP26 BAC. The insertion was confirmed via restriction digestion. The kanamycin cassette was removed from correct clones via the induction of the restriction enzyme *I-SceI* and *red* recombinase with 1% arabinose and 15 min of incubation at 42°C, respectively. Clones were confirmed via restriction digestion, and the sequencing of a PCR product of prW902 and prW429 validated the introduced modifications. Furthermore, the resulting BAC was subjected to next-generation sequencing (NGS). The recombinant BACs HSV1(17^+^)Lox-CheVP26 and HSV1(17^+^)Lox-CheVP26-pUL36ΔDUB were reconstituted via transfection into BHK cells.

### Preparation of virus stocks.

Viral stocks were generated in BHK or, for the complementation studies, in RSC-HAUL36 cells. Briefly, 10 to 40 dishes (15 cm) of cells were infected at confluence using a MOI of 0.005 to 0.01 PFU/cell and maintained with 2% FBS the full cytopathic effect had developed. Cells were harvested via scraping and pooled. Extracellular virus (ECV) and cells were separated via 10 min of centrifugation at 3,000 × *g*. The ECV was collected, and the cells were subjected to three freeze-thaw cycles to release cell-associated virus (CAV). The CAV was recovered via centrifugation (3,000 × *g*, 10 min, 4°C) to remove cellular debris. Pooled ECV and CAV were concentrated at 25,000 × *g* for 2 h and resuspended overnight in PBS on ice. The mixture was dounced 30× on ice to completely resuspend the pellet and was then layered onto a continuous 5 to 15% polysucrose 400 gradient. Heavy particles were separated at 25,000 × *g* at 4°C for 2 h, collected by pipette aspiration, filled up with ice-cold PBS, concentrated at 25,000 × *g* (4°C, 2 h), aliquoted, shock frozen in liquid nitrogen, and stored at −80°C. The virus aliquots were used only once.

### Virus titration.

The viruses used in this study were titrated on the cell line used for each experiment, using the exact downstream assay inoculation protocol. Cells were infected at confluence and overlaid with DMEM containing 2% FBS and 0.5% carboxymethylcellulose. Plaques were quantified 2 to 4 days postinfection (dpi) after formalin fixation and crystal violet (0.1%) staining.

### Growth curves.

Untreated cells were seeded 24 h prior to infection. Cells were infected at confluence for 1 h using a MOI of 0.001 (multistep) or MOI of 10 (single-step yield) and maintained in DMEM containing 2% FBS until complete CPE was observed. Total virus was harvested in 24 h intervals, and the yields were determined via plaque assays on Vero cells. Total virus was released by freeze-thawing the samples three times and removing the debris using a short spin (3,000 × *g*, 10 min, 4°C).

For growth curves with IFN treatment, Vero cells (60 to 80% confluent, seeded 24 h earlier), were treated with 500 IU/mL IFN-α2a (henceforth IFN-α) or with a combination of 500 IU/mL IFN-α and 100 IU/mL IFN-γ. Cells were pretreated for 16 to 20 h prior to inoculation and then infected at a MOI of 0.001 (multistep) and cultured for up to 5 dpi with samples harvested at 24 h intervals (cells scraped into medium) or infected at a MOI of 10 (single-step) and harvested at 24 hpi. MOIs were increased 2-fold for IFN-α and 10-fold (single step) or 100-fold (multistep) for the IFN-α/γ cotreatment. Total yields were determined via plaque assays on untreated Vero cells.

### Measuring viral DNA replication.

Vero cells were seeded in 24-well plates 24 h prior to IFN treatment. Cells were treated with a combination of 500 IU/mL IFN-α and 100 IU/mL IFN-γ and then infected with a MOI 1 or 10 (titer from untreated Vero) for 1 h. At 6 hpi, the medium was aspirated, and the cells were lysed with 400 μL DNA lysis buffer. The DNA was purified according to the manufacturer’s instructions (Zymo Research, number D3020). DNA was eluted in 20 μL and diluted 4 to 10-fold, and 2.5 μL were used for quantitation, using a PrimeTime Probe-based quantitative PCR master mix (Integrated DNA Technologies) with the primer/probe combinations shown in Table 2 under a standard 2-step PCR protocol.

**TABLE 2:** Primers used for quantitative PCR measurements of viral DNA

	Primer ID	Sequence
HSV-1	prW1545	5′-CATCACCGACCCGGAGAGGGAC-3′
	prW1546	5′-GGGCCAGGCGCTTGTTGGTGTA-3′
	UL30 probe	5′-[FAM]CCGCCGAACTGAGCAGACACCCGCGC[BHQ]-3′
Vero	prW1409	5′-CCCGATGGCCAGGTCA-3′
	prW1411	5′-GGTAGTTTCATGGATGCCACAG-3′
	Vero β-actin probe	5′- [HEX]CCATTGGCAATGAGCGG[BHQ1]-3′

### Plaque reduction assays.

Cells were seeded in 6-well plates to a density of 60 to 80% (24 h after seeding). The cells were treated with IFN-α (SK-N-SH, 25 IU/mL; RPE-1, 50 IU/mL; HaCaT, 100 IU/mL; Vero, 100 or 500 IU/mL) or a combination of IFN-α (500 IU/mL) and IFN-γ (100 IU/mL) for 16 to 20 h prior to inoculation. A single inoculum providing 100 to 200 PFU to each well was prepared for all of the IFN-α conditions. For the IFN-α/γ cotreatment, the inocula were serially diluted to give rise to 10^4^, 10^3^, or 10^2^ plaques. Besides these modifications, the assay functioned like a standard plaque assay. The cells were inoculated for 1 h with periodic agitation. To prevent the spread of extracellular virus, the cells were overlaid with 2% FBS and 0.5% carboxy-methylcellulose containing medium (+/− IFN). The cells were fixed with formalin at 2 dpi (untreated) or 4 dpi (IFN-treated), and stained with crystal violet. The plaque ratio was determined by dividing the number of plaques obtained from the IFN-treated cells by the mean number of plaques obtained from the untreated cells. For the IFN-α/γ combination treatment, the opposite ratio was determined (fold inhibition of plaque formation compared to untreated).

### Determination of plaque size.

Fixed and stained plaque assays of Vero cells were used to determine plaque size (from the plaque reduction assay). For each condition, multiple images of multiple wells were taken at hardware predefined positions using the EnSight plate reader 96-well imaging plug-in (*n* = 2 for IFN-α and IFN-α/γ). The plaque area was measured with the Fiji software package by manually outlining all visible plaques and using the Measure plug-in. A minimum of 32 plaques were counted for each replicate, and each plaque is represented in the corresponding graph. All analysis was done using GraphPad Prism 7. A two-way analysis of variance (ANOVA) was used to assess the difference between parental and mutant viruses and to compare treatment pairs (interaction).

### In-solution digestion prior to mass spectrometric (MS) analysis.

To analyze virion components, 20 μL aliquots from three separate gradient purified virion preparations were processed in parallel. Sample processing was performed as previously described (94). Proteins in Laemmli sample buffer (without β-mercaptoethanol) were initially incubated at 95°C for 3 min and then reduced in 50 mM DTT for 10 min at 70°C and alkylated with 120 mM iodoactamide for 20 min at room temperature in the dark. Protein precipitation was performed overnight at −20°C with a 4-fold volume of acetone. Pellets were washed four times with acetone at −20°C. Precipitated proteins were dissolved in 100 μL of 4 M urea in 100 mM of ammonium bicarbonate and digested with 0.25 μg of Lys-C (Wako) for 2 h at 30°C, followed by overnight digestion with 0.25 μg of trypsin at 37°C. Prior to the trypsin digestion, the samples were diluted to 2 M urea by adding 100 μL of 100 mM ammonium bicarbonate. The peptides were desalted using C-18 Stage Tips. Each Stage Tip was prepared with three discs of C-18 Empore SPE Discs (3M) in a 200 μL pipette tip. The peptides were eluted with 60% acetonitrile in 0.3% formic acid and dried in a vacuum concentrator (Eppendorf). The peptides were dissolved in 2% acetonitrile/0.1% formic acid prior to nanoLC-MS/MS analysis.

### TMT-labeling.

The peptides were dissolved in 20 μL of 10 mM HEPES buffer (pH 8.2). 5 μL (0.1 mg) of TMT Label Reagent (TMTsixplex Isobaric Label Reagent Set, Thermo Scientific) dissolved in anhydrous actonitrile was added to the sample. Incubation was performed for 90 min at room temperature at 300 rpm. To quench the reaction, 2 μL of a 0.06 mg/μL hydroxylamine solution in water was added and incubated for 5 min at 300 rpm at room temperature. The samples prepared with 6 different TMT labels (126 to 131) were pooled and cleaned up with Stage Tips as described above. The dried peptides were dissolved in 2% acetonitrile/0.1% formic acid prior to nanoLC-MS/MS analysis.

### NanoLC-MS/MS analysis.

NanoLC-MS/MS analyses were performed on an Orbitrap Fusion (Thermo Scientific) equipped with a PicoView Ion Source (New Objective) and coupled to an EASY-nLC 1000 (Thermo Scientific). Peptides were loaded on a trapping column (2 cm x 150 μm ID, PepSep) and separated on capillary columns (30 cm × 150 μm ID, PepSep) that were both packed with 1.9 μmC18 ReproSil and separated with a 45 min linear gradient from 3% to 30% acetonitrile and 0.1% formic acid with a flow rate of 500 nl/min. MS, MS2, and MS3 scans were acquired in the Orbitrap analyzer with a resolution of 60,000. For MS2 spectra, HCD fragmentation with a stepped collision energy of 35%, 40%, 45% was applied. For MS3 spectra, HCD fragmentation with a collision energy of 55% was applied. A top speed, data-dependent MS/MS method with a fixed cycle time of 3 s was used. Dynamic exclusion was applied with a repeat count of 1 and an exclusion duration of 30 s. Singly charged precursors were excluded from selection. The minimum signal threshold for precursor selection was set to 50,000. Standard AGC targets were set for MS and MS2 scans. For the MS3 scans, a customized AGC target of 300% was set. EASY-IC was used for internal calibration.

### MS data analysis.

A database search was performed against the UniProt Herpes Virus database (December 21, 2020, UP000009294, 73 entries) using the PEAKS Xpro software package (Bioinformatics Solutions, Inc.) with the following parameters: peptide mass tolerance, 8 ppm; MS/MS mass tolerance, 0.02 Da; enzyme, trypsin with 2 missed cleavage sites; variable modifications: oxidation (M) and pyro-glu from Q; fixed modifications: TMTsixplex and carbamidomethylation (C). The UniProt *Cricetulus griseus* database (October 26, 2020, UP000001075, 23,885 entries) was used as the contaminant database. Results were filtered to 1% PSM-FDR via the target-decoy approach. Quantification was performed with 15 ppm of the quantification mass tolerance. Isotope purity correction for the TMT marker fragments was performed according to information from the manufacturer.

### IFN recovery assay.

To confirm the results of the plaque reduction assay, we adapted the experiment to a 96-well format. A total of 6,000 Vero cells were seeded per well to obtain approximately 60% confluence at the start of the IFN-α treatment. After 24 h, the regular growth medium was replaced with a medium lacking (mock) or containing 500 IU/mL of IFN-α. After 16 to 20 h, the media were removed, and the cells were washed with serum-free medium and infected with 50 μL of serum-free medium containing 1.5 to 3 PFU/well of HSV1(17+)Lox-CheVP26 or HSV1-CheVP26-UL36.C65A. The virus dose was calculated based on a Poisson distribution to generate a phenotypic window between the parental and DUB mutant viruses, assuming a roughly 2-fold drop in infectivity, which we observed in the plaque reduction assay. After 2 h, 150 μL/well of mock or IFN-α-containing media were added to yield an IFN-α concentration of 500 IU/mL. After 6 days, half of the medium was transferred to untreated Vero cells cultured in 96-well plates and incubated for another 4 days. Infected wells were scored via fluorescence microscopy (mCherry) after fixation and crystal violet staining. The ratio of infected wells for each condition compared to those of the untreated samples was formed as before (+/− IFN-α) and plotted. Statistical testing was performed essentially as for the plaque reduction assay.

### Statistical analysis.

All significance testing was done using the One-Way ANOVA or Two-Way ANOVA plug-ins of GraphPad Prism 7. No corrections for multiple comparisons were conducted.

### Immunofluorescence microscopy.

Vero cells were seeded in 12-well IBIDI chamber slides at 10,000 cells per well (60% confluence the next day) and treated, after 24 h, with 500 IU/mL IFN-α or with a combination of 500 IU/mL IFN-α and IFN-γ for 16 to 20 h. Virus inocula were serially diluted 10-fold from a MOI of 10 to a MOI of 0.01. Cells were inoculated for 1 h, and then the medium was exchanged (+/− IFN). IFN-treated and untreated cells were infected with the same inoculum for each MOI. The cells were fixed at 24 hpi and stained with DAPI, anti-ICP4 (Santa Cruz), and anti-mouse Alexa Fluor 488 (Life Technologies). Infected cells were imaged using DAPI, FITC, and Texas Red filter sets on a Leica DMi 8 inverted microscope.
